# Suppressing microRNA-29c promotes biliary atresia-related fibrosis by targeting DNMT3A and DNMT3B

**DOI:** 10.1186/s11658-018-0134-9

**Published:** 2019-03-11

**Authors:** Jian-yao Wang, Hao Cheng, Hong-yan Zhang, Yong-qin Ye, Qi Feng, Zi-min Chen, Yue-lan Zheng, Zhou-guang Wu, Bin Wang, Jun Yao

**Affiliations:** 1 Department of General Surgery, Shenzhen Children’s Hospital, Shenzhen, 518026 Guangdong Province China; 2 Graduate School of China Medical University, Shenzhen, 110122 Liaoning Province China; 3 Department of Gastroenterology, Jinan University of Medical Sciences, Shenzhen Municipal People’s Hospital, Shenzhen, 518020 Guangdong Province China

**Keywords:** Biliary atresia, Epithelial–mesenchymal transition, MiR-29c, Fibrosis, DNMT3A, DNMT3B

## Abstract

This study was designed to investigate the potential role of microRNA-29c (miR-29c) in biliary atresia-related fibrosis. The expression of miR-29c was determined in 15 pairs of peripheral blood samples from infants with biliary atresia (BA) and infants with non-BA neonatal cholestasis using quantitative real-time PCR. EMT was established by induction with TGF-β1 in HIBEpiC cells. MiR-29c was inhibited by lipofectamine transfection. The expressions of proteins related to epithelial–mesenchymal transition (EMT), i.e., E-cadherin, N-cadherin and vimentin, were determined using quantitative real-time PCR and western blotting. Direct interaction between miR-29c and DNMT3A and DNMT3B was identified using a luciferase reporter assay. The expressions of DNMT3A and DNMT3B were suppressed by treatment with SGI-1027. Patients with BA showed significantly lower miR-29c levels in peripheral blood samples than the control subjects. In vitro, TGF-β1-induced EMT significantly decreased the expression of miR-29c. Downregulation of miR-29c had a promotional effect on BA-related fibrosis in HIBEpiC cells, as confirmed by the decrease in E-cadherin and increase in N-cadherin and vimentin levels. MiR-29c was found to target the 3’UTR of DNMT3A and DNMT3B and inhibit their expression. Suppression of DNMT3A and DNMT3B reversed the effects of miR-29c downregulation on BA-related fibrosis in HIBEpiC cells. These data suggest that BA-related fibrosis is closely associated with the occurrence of EMT in HIBEpiC cells. MiR-29c might be a candidate for alleviating BA-related fibrosis by targeting DNMT3A and DNMT3B.

## Background

Biliary atresia (BA) is a devastating fibrotic disorder of unknown etiology. It impacts the extrahepatic biliary tree of newborns, leading to progressive fibrotic biliary duct obstruction along with the development of biliary cirrhosis [1, 2]. The disease is characterized by prolonged cholestatic jaundice during the neonatal period [3]. From 1 in 8000 to 12,000 new BA cases are projected to occur globally per year [4]. Kasai portoenterostomy (KPE), liver transplantation or the two approaches together have proven effective in the treatment of BA [5]. The causes and pathogenesis of the cellular inflammation and progressive fibrosis that contribute to the progression of BA are well documented [5].

Epithelial–mesenchymal transition (EMT) was recently shown to be involved in the formation of fibrogenic myofibroblasts in liver fibrosis and to be associated with occurrences of biliary atresia and primary biliary cirrhosis [6]. Previous developmental investigations with adult organisms and cell cultures have yielded various growth and differentiation factors, such as TGF-β, and other growth factors that act through receptor tyrosine kinases. All of these can induce and regulate EMT [7]. Although BA management has been extensively studied, the pathological mechanisms behind the related EMT processes are poorly understood.

MicroRNAs (miRNAs) are an abundant, evolutionarily conservative class of small (20- to 23-nucleotide) non-coding RNAs [8]. They are known to play a key role in silencing targeted genes by inducing mRNA degradation, translational repression and destabilization [9]. During liver development, the expressions of 38 miRNAs obviously change. MiR-30a and miR-30c are specifically expressed in cholangiocytes [10, 11]. Nakamura et al. showed that depletion of miR-30a impaired bile duct formation in zebra fish and proposed its importance in regulating biliary differentiation [11].

Two members of miR-29 family, miR-29a and miR-29b1, were recently shown to be upregulated in an established mouse model of BA. They act as regulators of BA pathogenesis by targeting Igf1 and Il1RAP [12]. Zhang et al. [13] found that amplification of miR-29c could suppress the EMT progression induced by Sp1/TGF-β in lung cancer. MiR-29c is also reported to mediate EMT due to GNA13 and PTP41 modulation of β-catenin signaling pathways in human colorectal cancer [14].

Accumulating evidence has implicated epigenetic regulation in the pathogenesis of BA [11]. The occurrence of DNA methylation at the position of the cytosine ring in the CpG dinucleotide is essential for epigenetic modification of the human genome [15]. DNA methyltransferases, including DNMT1, DNMT3A and DNMT3B, are responsible for adding a methyl group to cytosine residues, and thus affect gene silencing, chromatin structure, disease progress and development [16]. Yang et al. [17] proposed a role for miR-29b and miR-142-5p in facilitating the etiology of BA via DNMTs regulation of IFN-γ. Pandi et al. [18] confirmed that miR-29c plays a critical role in mediating ischemic brain damage though targeting DNMT3a. Based on previous studies that indicated an association of miR-29 with EMT processes and DNMTs, we predict that miR-29c is involved in the etiology of BA.

In this study, we investigated the expression patterns of miR-29c in peripheral blood samples from BA patients. We treated HIBEpiC cells with TGF-β to induce EMT, then examined the role of miR-29c in the regulation of EMT biomarkers and in specific mechanisms that might be related to BA. This study may provide a candidate target for new therapeutic strategies against BA.

## Materials and methods

### Sample collection and processing

The subjects were fifteen 30- to 90-day old infants with BA and fifteen age-matched infants with non-BA neonatal cholestasis. Diagnosis was with a laparoscopic bile duct exploration at the Shenzhen Children’s Hospital in Guangdong, China. All the BA patients underwent a hepatoportoenterostomy (Kasai) surgery between the ages of 30 and 90 days. Their parents gave written informed consent for their children to be involved in the study prior to the surgery. This study was performed in accordance with the relevant guidelines and regulations approved by the ethics committee of the Shenzhen Children’s Hospital in Guangdong, China.

Peripheral blood samples were collected from all the subjects and kept for about 1 h at room temperature. These blood samples underwent centrifugation at 820×g for 10 min at 4 °C to obtain serum, followed by further centrifugation at 16,000×g for 10 min at 4 °C to completely remove cell debris. The supernatant serum was stored at − 20 °C until analysis.

### Cell treatments and transfection

The human normal intrahepatic biliary epithelial cell line (HIBEpiC) was purchased from the BeNa Culture Collection and cultured in RPMI 1640 medium (Gibco) supplemented with 10% fetal bovine serum (FBS, Gibco) and 1% penicillin/streptomycin. 293 T cells were obtained from the Cell Bank of the Chinese Academy of Science and cultured in Dulbecco’s modified Eagle medium (DMEM). The two cell lines were maintained in a humidified incubator with an atmosphere of 5% CO_2_ at 37 °C. Subsequently, the HIBEpiC cells were induced into EMT using a conditioned medium containing TGF-β1 (10 ng/ml) for 48 h, as previously described [19].

The oligonucleotides of the miR-29c mimics, miR-29c inhibitor and their corresponding negative controls (mimics NC and inhibitor NC, respectively) were synthesized at Genepharma Co., Ltd. For cell transfection, HIBEpiC cells were seeded in six-well plates and grown to 80% confluence, followed by transfection with miR-29c inhibitor using Lipofectamine 2000 Reagent (Invitrogen) according to the manufacturer’s protocol. For DNMT3A and DNMT3B suppression, HIBEpiC cells transfected with miR-29c inhibitor were treated with DNMT3A/DNMT3B inhibitor SGI-1027 (Selleckchem) for 36 h.

### Quantitative real-time PCR

Total RNA was isolated from the serum sample or cells using a MiniBEST Universal RNA Extraction Kit (Takara) according to the manufacturer’s instructions. The cDNA was synthesized from 1 μg total RNA using a miScript Reverse Transcription kit (Qiagen) for miR-29c or PrimeScript RT Reagent Kit (Takara) for related genes. For the detection of miR-29c expression, real-time PCR was performed in triplicate using a TaqMan miRNA assay on an ABI 7500 Fast Real-Time PCR System (Applied Biosystems) with U6 as an internal control, according to the manufacturer’s instructions. The relative quantification of gene expression was performed using a SYBR Green PCR kit (Takara) on an ABI 7500 Fast Real-Time PCR System (Applied Biosystems) with GAPDH as an internal control, following the manufacturer’s instructions. The primer sequences were:

- miR-29c forward: 5’-TAGCACCATTTGAAAT-3′; reverse: 5’-GTGCAGGGTCCGAGGT-3′;
- U6 forward: 5’-CTCGCTTCGGCAGCACA-3′; reverse: 5′- AACGCTTCACGAATTTGCGT-3′;
- DNMT3A forward: 5’-GCGCCTCAGAGCTATTACCC-3′; reverse: 5’-GCAGCCATTTTCCACTGCTC3’;
- DNMT3B forward: 5’-ACAGGACTTGACAGGCGATG-3′; reverse: 5’-TGGGCTTTCTGAACGAGTCC-3′;
- E-cadherin forward: 5′ –GAGAAACAGGATGGCTGAAGG-3′; reverse: 5’-TGAGGATGGTGTAAGCGATGG-3′;
- N-cadherin forward: 5’-ATGAAAGACCCATCCACGC-3′; reverse: 5’-CCTGCTCACCACCACTAC-3′);
- Vimentin forward: 5’-AGTCCACTGAGTACCGGAGAC-3′; reverse: 5’-CATTTCACGCATCTGGCGTTC-3′;
- GAPDH forward: 5’-GAGTCAACGGATTTGGTCGT-3′; reverse: 5’-GACAAGCTTCCCGTTCTCAG-3′.

All measurements were repeated in triplicate. The relative levels of miR-29c or mRNA were calculated using the comparative 2^-∆∆Ct^ method.

### Western blotting analysis

Western blotting was performed according to standard procedures using enhanced chemiluminescence detection. Briefly, total proteins were extracted from cells using RIPA lysis buffer (Thermo Fisher Scientific). Equal amounts of protein were separated on sodium dodecyl sulfate–polyacrylamide gels and then polyvinylidene fluoride membrane (Millipore). After blocking with 5% skim milk, the membranes were incubated with the primary antibodies for DNMT3A, DNMT3B, E-cadherin, N-cadherin, vimentin and GAPDH (Abcam), followed by incubation with corresponding horseradish peroxidase-coupled secondary antibody (Beyotime). The signals were detected with enhanced chemiluminescence reagents (Pierce). GAPDH was used as an internal control.

### MiR-29c target prediction

TargetScan (http://www.targetscan.org), MiRanda (http://www.microrna.org/microrna/home.do) and PicTar (http://pictar.mdc-berlin.de/) were used to predict possible target genes of miR-29c and conserved sites bound by the seed region of miR-29c in silico.

### Luciferase reporter assay

To determine whether DNMT3A or DNMT3B was a direct target of miR-29c, DNMT3A or DNMT3B mRNA 3’UTR fragments containing the putative miR-29c-binding sequences were amplified using PCR and cloned into pmirGLO plasmids (Promega Corporation): Luc-DNMT3A-wt and Luc-DNMT3B-wt, respectively. The mutant of the DNMT3A or DNMT3B 3’UTR sequence containing the putative binding site of miR-29c was synthesized via PCR using site-directed mutagenesis with a QuickChange II XL Site-Directed Mutagenesis Kit (Stratagene). The mutants were also cloned into pmirGLO plasmids: Luc-DNMT3A-mut and Luc-DNMT3B-mut, respectively.

For the luciferase reporter assay, 293 T cells were seeded into 24-well plates and co-transfected with 0.5 μg of constructed reporter plasmids (Luc-DNMT3A or DNMT3B-wt and Luc-DNMT3A or DNMT3B-mut) together with miR-29c mimics or mimics NC at a final concentration of 50 nM using Lipofectamine 2000 according to the manufacturer’s instructions. After 48 h, luciferase activity was measured using the Dual-Luciferase Reporter Assay system (Promega) according to the manufacturer’s protocol.

### Statistical analysis

All statistical analyses were performed using SPSS 17.0 statistical software. All quantitative data are expressed as the mean ± standard deviation (SD) from at least three separate experiments. Student’s t test was used to calculate the differences between the two study groups. Pairwise multiple comparisons were performed with one-way ANOVA (two-sided). Differences were considered statistically significant at *p* < 0.05.

## Results

### Lower plasma levels of miR-29c were characteristic for BA patients

The expression levels of miRNAs recently emerged as potential biomarkers for various pathological conditions. To evaluate the role of miR-29c in BA pathogenesis, its expression was determined in peripheral blood samples from BA patients using quantitative real-time PCR analysis.

**Fig. 1:**
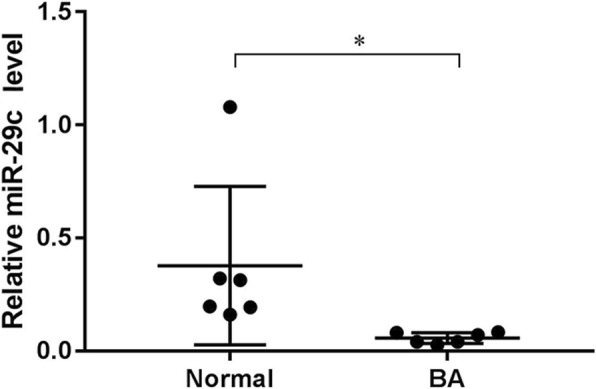
Expression of miR-29c in the peripheral blood of infants with biliary atresia. Normal subjects (Normal): *n* = 15. Biliary atresia subjects (BA): *n* = 15; *p < 0.05 vs. Normal

The expression of miR-29c was significantly downregulated in BA patients compared with the controls (*p* < 0.05; Fig. 1). These results indicate that dysregulation of miR-29c might be associated with the progression of BA.

### TGF-β1-induced EMT significantly decreased the expression of miR-29c in HIBEpiC cells

BA is characterized as a fibrous obstruction. MiRNAs are known to be involved in fibrotic process. To investigate the functional role of miR-29c in BA, HIBEpiC cells were treated with a major inducer of EMT, TGF-β1, to create an in vitro BA cell model.

**Fig. 2:**
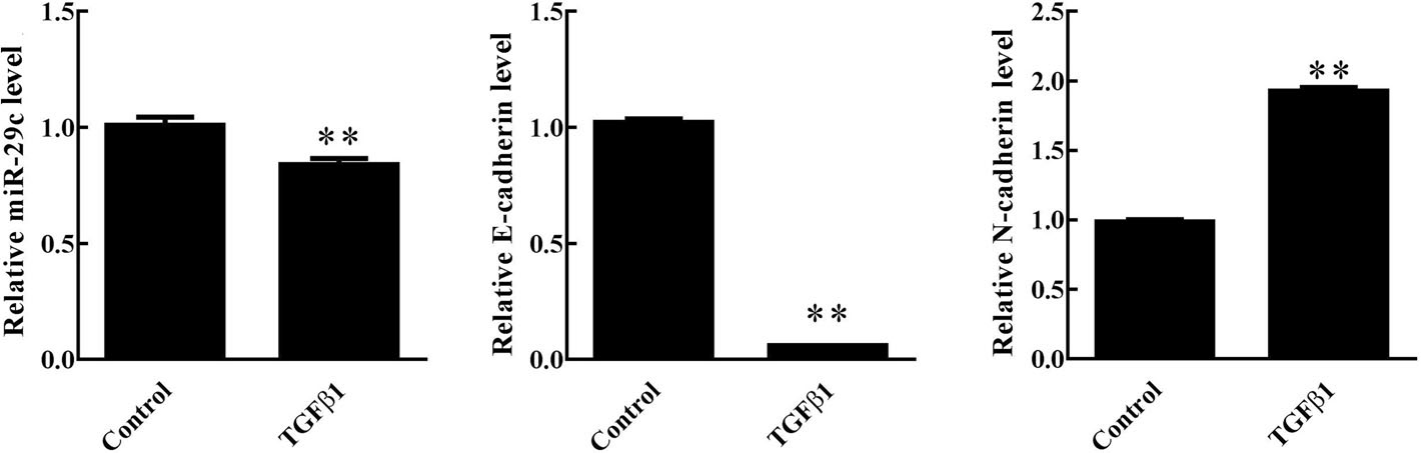
Changes in the expressions of miR-29c, E-cadherin and N-cadherin in HIBEpiC cells treated with TGFβ1. **p < 0.05 vs. the control

The expression of miR-29c in HIBEpiC cells significantly decreased after stimulation with TGF-β1 (*p* < 0.01; Fig. 2). The HIBEpiC cells also showed significantly lower E-cadherin expression (*p* < 0.01), but higher N-cadherin expression (*p* < 0.01) after TGF-β1 treatment. These data indicate that miR-29c might play an important role in BA pathogenesis by regulating fibrous obstruction.

### Downregulation of miR-29c promoted BA-related fibrosis in HIBEpiC cells

**Fig. 3:**
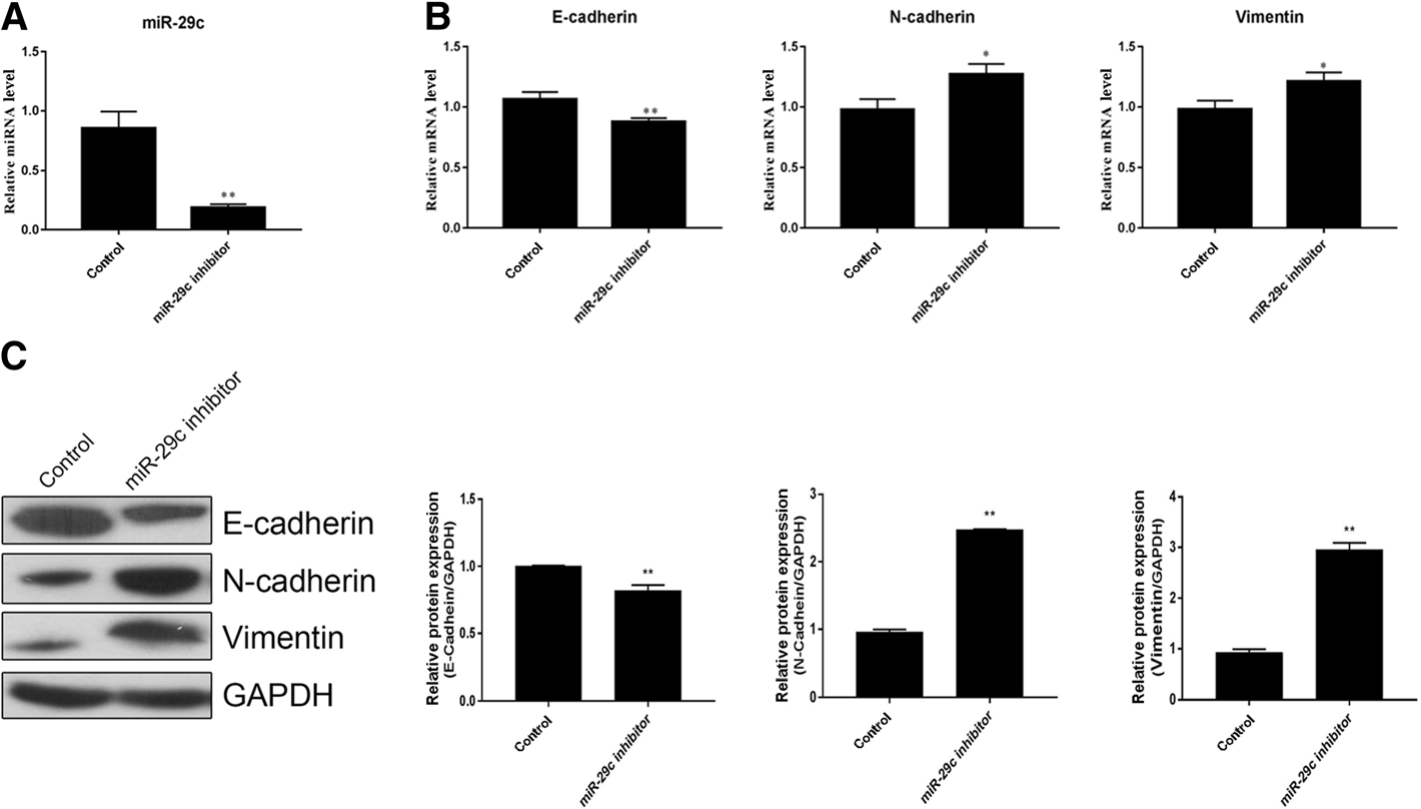
Downregulating miR-29c promoted BA fibrosis in HIBEpiC cells. **a** Downregulation of miR-29c was confirmed using qPCR. **b** The expressions of E-cadherin, N-cadherin and vimentin were determined using qPCR. **c** Western blot analysis of E-cadherin, N-cadherin and vimentin levels

As miR-29c was downregulated in TGF-β1-induced EMT, we speculated that miR-29c might suppress BA-related fibrosis in vitro. To validate our hypothesis, HIBEpiC cells were transfected with an miR-29c inhibitor to downregulate the expression of miR-29c. This downregulation was confirmed using quantitative real-time PCR (*p* < 0.01; Fig. 3a). We further observed that downregulation of miR-29c significantly inhibited the expression of E-cadherin and increasing the expressions of N-cadherin and vimentin. The expressions were determined using quantitative real-time PCR (*p* < 0.05 and *p* < 0.01, respectively; Fig. 3b) and western blot analysis (Fig. 3c). This evidence suggests that miR-29c might be a potential candidate for ameliorating BA.

### Negative regulation of DNMT3A and DNMT3B expression by miR-29c in HIBEpiC cells

**Fig. 4:**
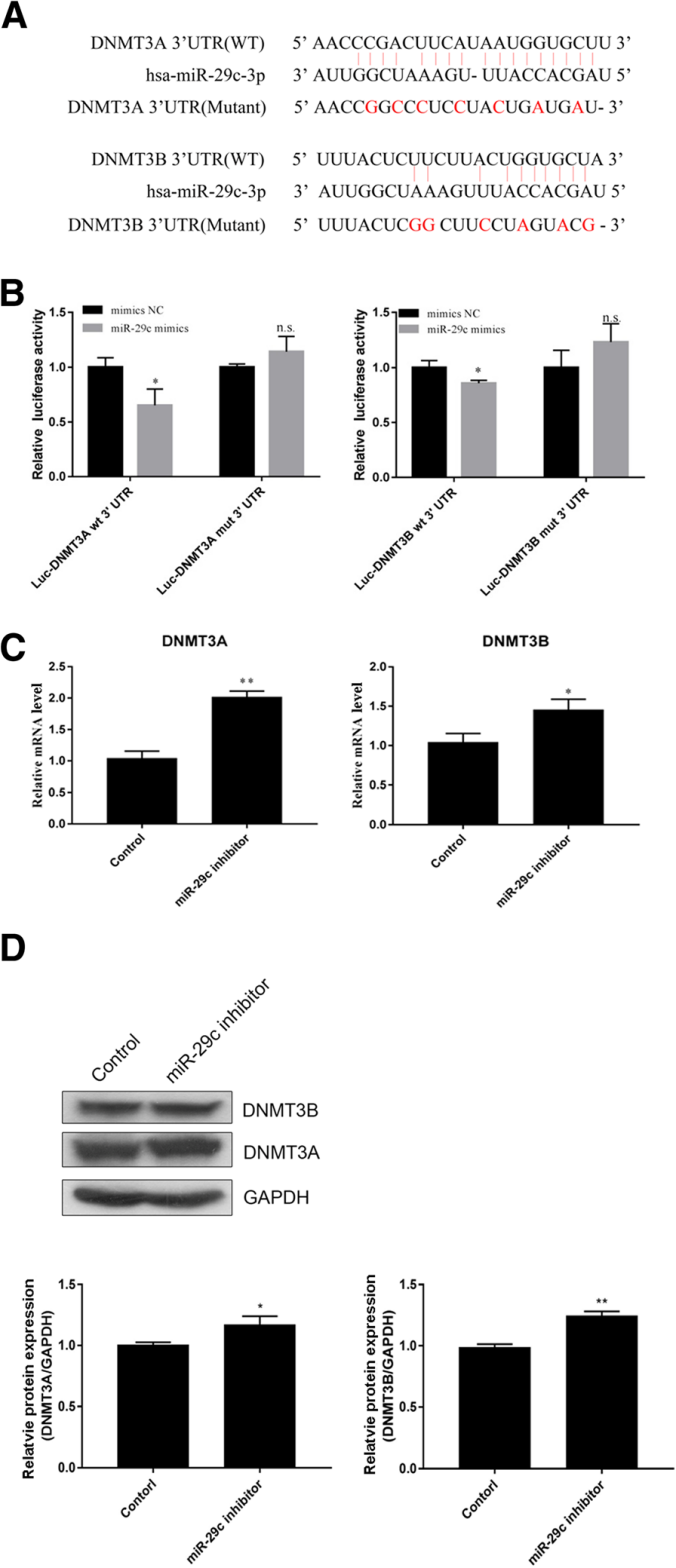
MiR-29c targeted DNMT3 and DNMT3B. **a** The site on DNMT3A and DNMT3B targeted by miR-29c was predicted using miRNA.org software. **b** Confirmation of miR-29c targeting of DNMT3A and DNMT3B was obtained using the luciferase reporter assay. *p < 0.05 vs. the control. **c** and **d** The DNMT3A and DNMT3B mRNA and protein levels in HIBEpiC cells transfected with an miR-29c inhibitor was measured using qPCR and western blot assays. *p < 0.05 vs. the control; **p < 0.01 vs. the control

A bioinformatic analysis was performed to predict the potential target genes of miR-29c and elucidate the molecular mechanism underlying miR-29c regulation of BA. As DNA methylation is involved in the pathogenesis of BA, DNMT3A and DNMT3B, which participate in DNA methylation, were selected as the targeted genes of miR-29c. As shown in Fig. 4a, miR-29c could bind to the 3’UTR of DNMT3A and DNMT3B. This was confirmed via luciferase reporter assay, which showed that co-transfection with an miR-29c mimic significantly reduced DNMT3A- and DNMT3B-driven luciferase activity in 293 T cells (*p* < 0.05; Fig. 4b). HIBEpiC cells transfected with an miR-29c inhibitor also showe higher DNMT3A and DNMT3B mRNA levels (*p* < 0.05 and *p* < 0.01 respectively; Fig. 4c) and protein levels (Fig. 4d). These results suggest that miR-29c binds the 3’UTR of DNMT3A and DNMT3B and negatively regulates their expression.

### Suppression of DNMT3A and DNMT3B reversed the effects of miR-29c downregulation on BA-related fibrosis in HIBEpiC cells

**Fig. 5:**
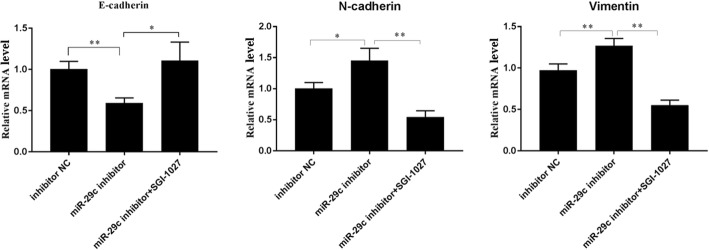
miR29c promoted BA in HIBEpiC by upregulating the expressions of DNMT3A and DNMT3B. *p < 0.05 vs. the control; **p < 0.01 vs. the control

To further investigate whether miR-29c regulated BA by targeting DNMT3A and DNMT3B, the expressions of DNMT3A and DNMT3B were inhibited in HIBEpiC cells by treatment with SGI-1027 after transfection with an miR-29c inhibitor. The quantitative real-time PCR results (Fig. 5) show that SGI-1027 treatment could significantly alleviate the suppressive effect of the miR-29c inhibitor on E-cadherin (*p* < 0.05, *p* < 0.01) expression and enhance the effects of the miR-29c inhibitor on the expressions of N-cadherin (*p* < 0.05, *p* < 0.01) and vimentin (*p* < 0.01) in HIBEpiC cells. Collectively, these results further suggest that miR-29c could suppress BA by directly downregulating DNMT3A and DNMT3B.

## Discussion

MiRNAs are considered to be a key regulatory layer in fundamental biological processes, including differentiation, proliferation, embryogenesis, development and cell fate [20]. Several miRNAs are reported to be involved in various fibrosis contexts. The miR-199 and miR-200 family could be implicated in liver fibrosis: a study revealed thatmiR-199 and -200 family members (miR-199a, miR-199a*, miR-200a and miR-200b) were upregulated in human and mouse liver biopsy specimens [21]. Liu et al. [22] reported that intravenous administration of miR-21 antisense probes attenuated lung fibrosis in a mouse model by regulating the profile of Smad7, which is an inhibitory Smad. MiR-24 is a major controller of genes associated with cardiac fibrosis [23]. Considerable recent evidence has also supported the regulation of the miR29 gene cluster in fibrosis of the liver, kidneys, lungs and heart, although the role of miR-29c in BA has not yet been defined [24].

This study determined the miR-29c expression patterns in peripheral blood samples from BA patients and healthy newborn infants. We identified that miR-29c is a downstream mediator of TGF-β-induced EMT in HIBEpiC cells, and that the gene has an inhibitory effect on N-cadherin and imventin and a stimulatory effect on E-cadherin. We have also shown that low expression of miR-29c-induced fibrosis is linked with high levels of DNMT3B and DNMT3A, two direct targets of miR-29c.

Increasingly, aberrant expression of circulating miRNAs is found to mirror events at the histological and molecular levels in various human organs [20]. We have confirmed that low expression of miR-29c is a dangerous event in BA and a potential biomarker for poor prognosis.

EMT was recently established as a key pathological pathway that increases the number of fibrogenic cells. It is a source of fibroblasts in some fibrotic organs [6]. The expression of E-cadherin is decreased during epithelial cell acquisition as a mesenchymal phenotype [25]. The presence of vimentin and a switch from E- to N-cadherin have been shown to be hallmarks of EMT [26].

In parallel with the occurrence of EMT in BA, accumulating evidence supports TGF-β as a critical mediator of the progression of fibrosis [27]. TGF-β appears to be implicated in hepatic fibrogenesis, where it influences the phenotypic and functional plasticity of hepatic stellate cells [27]. Suggestive evidence for the involvement of TGF-β in the progression of renal and liver fibrosis was obtained by regulating both canonical and non-canonical pathways [27, 28].

In our study, we observed reduced miR-29c and E-cadherin and increased N-cadherin expressions in HIBEpiC cells after TGF-β treatment. The miR-29c inhibitor generally exhibited a TGF-β-like phenotype. Upregulation of vimentin also occurred. These results suggest that miR-29c is a mediator of TGF-β and EMT signaling pathways, and that miR-29c downregulation by TGF-β could accelerate the progress of BA.

It is well known that a large number of protein-coding genes are targeted by one or more miRNAs [29]. Rooij et al. [30] pointed out that collagens, fibrilins and elastin, which are related to fibrosis, are targeted by miR-29. A previous study revealed that the reversion of the miR-29 family on abnormal methylation in lung cancer is mediated though regulation of DNMT3A and DNMT3B, two critical de novo DNA methyltransferases that are frequently upregulated in patients with idiopathic pulmonary fibrosis [31].

Here, we identified DNMT3A and DNMT3B as direct targets of miR-29c. Importantly, SGI-1027, a potent inhibitor of DNMT could significantly reverse the effects of miR-29c on E-cadherin, N-cadherin and vimentin.

Han et el. [32] detected the relationship between DNA methylation and miRNA levels in colorectal cancer HCT116 cells, and found that approximately 10% of miRNAs were influenced by DNA methylation. Downregulation of E-cadherin is a direct consequence of an increase in the methylation of the promoter CpG. On the other hand, miR-29c may be regulated by DNA methylation via an indirect mechanism. The transcription factors that modulate N-cadherin and vimentin may also be dysregulated by DNA methylation. In short, depression of miR-29c promotes the BA-related fibrosis that is induced by TGF-β by targeting DNMT3A and DNMT3B.

We found that miR-29c is decreased in peripheral blood samples of BA patients. TGF-β and miR-29c signaling pathways play a significant role in the etiology of BA. This study may open several approaches for fighting BA.

## Abbreviations

BA: Biliary atresia
EMT: Epithelial–mesenchymal transition
